# Protease-activated receptor 2 activation induces behavioural changes associated with depression-like behaviour through microglial-independent modulation of inflammatory cytokines

**DOI:** 10.1007/s00213-021-06040-1

**Published:** 2021-12-09

**Authors:** Serge Moudio, Ashleigh Willis, Karolina Pytka, Roua Abulkassim, Ros R. Brett, Jack F. Webster, Christian Wozny, Mark Barbour, Hui-Rong Jiang, David G. Watson, Josie C. van Kralingen, Scott M. MacKenzie, Michael Daniels, Barry W. McColl, Sandra Sossick, Hugh N. Nuthall, Trevor J. Bushell

**Affiliations:** 1 Strathclyde Institute of Pharmacy & Biomedical Sciences, University of Strathclyde, 161 Cathedral Street, Glasgow, G4 0RE UK; 2 Department of Pharmacodynamics, Faculty of Pharmacy, Jagiellonian University Medical College, Medyczna 9, 30-688, Krakow, Poland; 3 MSH Medical School Hamburg, Medical University, Am Kaiserkai 1, 20457 Hamburg, Germany; 4 Institute of Cardiovascular and Medical Sciences, University of Glasgow, 126 University Place, Glasgow, G12 8TA UK; 5 UK Dementia Research Institute at The University of Edinburgh, Edinburgh Medical School, The Chancellor’s Building, 49 Little France Crescent, Edinburgh, EH16 4SB UK; 6 Eli Lilly and Co. Ltd.8 Arlington Square, Bracknell, RG12 1WA UK

**Keywords:** Open-field test, Sucrose preference, Inflammation, Cytokines, Protease-activated receptor-2

## Abstract

**Rationale:**

Major depressive disorder (MDD) is a leading cause of disability worldwide but currently prescribed treatments do not adequately ameliorate the disorder in a significant portion of patients. Hence, a better appreciation of its aetiology may lead to the development of novel therapies.

**Objectives:**

In the present study, we have built on our previous findings indicating a role for protease-activated receptor-2 (PAR2) in sickness behaviour to determine whether the PAR2 activator, AC264613, induces behavioural changes similar to those observed in depression-like behaviour.

**Methods:**

AC264613-induced behavioural changes were examined using the open field test (OFT), sucrose preference test (SPT), elevated plus maze (EPM), and novel object recognition test (NOR). Whole-cell patch clamping was used to investigate the effects of PAR2 activation in the lateral habenula with peripheral and central cytokine levels determined using ELISA and quantitative PCR.

**Results:**

Using a blood–brain barrier (BBB) permeable PAR2 activator, we reveal that AC-264613 (AC) injection leads to reduced locomotor activity and sucrose preference in mice but is without effect in anxiety and memory-related tasks. In addition, we show that AC injection leads to elevated blood sera IL-6 levels and altered cytokine mRNA expression within the brain. However, neither microglia nor peripheral lymphocytes are the source of these altered cytokine profiles.

**Conclusions:**

These data reveal that PAR2 activation results in behavioural changes often associated with depression-like behaviour and an inflammatory profile that resembles that seen in patients with MDD and therefore PAR2 may be a target for novel antidepressant therapies.

**Supplementary Information:**

The online version contains supplementary material available at 10.1007/s00213-021-06040-1.

## Introduction

Major depressive disorder (MDD) is a major contributor to the global burden of disease (Miller and Raison 2016; Bhattacharya and Drevets 2017; WHO Mental Health Report 2020), and contributing to this burden is the significant number of patients that are treatment-resistant (TRD), whilst other sufferers experience relapses despite being on current therapies (Bhattacharya and Drevets 2017). Thus, it is clear that treatment of depression is an unmet clinical need and there is a profound need to improve our understanding of the aetiology of MDD so that new, better-targeted interventions can be developed (Pape et al. 2019). In particular, the role of inflammation in affective disorders, including depression, is currently an intense area of research with significant interest in inflammatory cytokines and their role in MDD (Felger 2019; Branchi et al. 2020). Indeed, studies have shown that patients with MDD, particularly those with TRD or who do not respond as well to current therapies, have increased serum levels of pro-inflammatory cytokines relative to healthy matched controls (Lamers et al. 2016; Köhler et al. 2017; Chamberlain et al. 2019; Felger 2019; Branchi et al. 2020), but how these elevated levels directly relate to depression is unclear. Hence, animal models of depression-like behaviour have been widely used to further our understanding of the aetiology of depression.

Several rodent models have been developed and proposed to induce depression-like behaviour (Nestler and Hyman 2010; Wang et al. 2017). Pathogen-induced depression-like behaviour, using the endotoxin lipopolysaccharide (LPS), is widely used to induce sickness-like behaviour (Dantzer 2017), with recent studies indicating that escalating LPS doses results in depression-like behavioural symptoms in addition to serum and brain inflammatory profiles similar to those seen in MDD (Krishna et al. 2016; Rodrigues et al. 2018; Wickens et al. 2018). However, the direct mechanism by which this occurs and whether peripheral inflammation is directly involved remains unclear. We have previously shown that protease-activated receptor 2 (PAR2) deletion results in impaired LPS-induced sickness behaviour (Abulkassim et al. 2016) and that PAR2 activation impairs synaptic transmission in the hippocampus (Gan et al. 2011), a brain region proposed to play a key role in depression (Malykhin and Coupland 2015; Boku et al. 2018). Hence, in the present study, we have built on our previous findings to show that the selective PAR2 activator, AC-264613 (AC; Gardell et al. 2008; Barry et al. 2010), crosses the blood–brain barrier (BBB) and induces behavioural changes similar to those observed in depression-like behaviour. In addition, AC-induced behavioural changes correlate with increased serum IL-6 levels and brain IL-1β mRNA, with these data being analogous to those reported previously in animal models of depression-like behaviour and in MDD (Bhattacharya and Drevets 2017; Dantzer 2017). These novel findings provide further evidence to our previous proposals that PAR2 plays a role in behavioural changes associated with depression-like behaviour and thus warrants further investigation as a novel target for future therapeutic interventions for the treatment of MDD.

## Materials and Methods

Detailed methods are described in Online Resource 1.

### Animals

All in vivo experimental procedures comply with ARRIVE guidelines and were in accordance with UK legislation (Animals (Scientific Procedures) Act 1986) and with approval by the University of Strathclyde Ethics Committee. All experiments were completed using C57BL/6 J mice (10–12 weeks old) group-housed (5–6 per cage) under standard conditions (21 ± 1 °C, 45–65% humidity, 12-h dark/light cycle [lights on 06:00]) in MB1 cages (45 × 28 × 13 cm) lined with grade 6 wood bedding and containing a plastic refuge and nesting material. Animals had free access to food and water with all behavioural procedures carried out between 09.00 and 17.00. Mice were randomly assigned to the treatment groups with the experimenter blinded to each group.

### Pharmacokinetic profiling of AC

*Initial LC–MS:* Male C57BL/6 J mice injected intraperitoneally (i.p.) with vehicle (*N* = 4, 0.9% saline solution including 1% Tween 80), AC (*N* = 8, 10 mg kg^−1^, Tocris, UK), or SLIGRL-NH_2_ (*N* = 8, 10 mg kg^−1^, Peptide Synthetics, UK) were killed by cervical dislocation at specific time points (0.5–1 h) postinjection with the brains immediately frozen and stored at − 80 °C until required. Liquid chromatography-mass spectrometry (LC–MS) was performed using an Orbitrap Exactive mass spectrometer (ThermoFisher, Hemel Hempstead, UK) coupled to a Dionex HPLC system (Dionex, Camberley, UK) with the elution ‘finger-print’ of AC (MW 400.1, elution time ≈16 min) and SLIGRL-NH_2_ (MW 658, elution time ≈16.5 min) initially determined using a known concentration. Original signal spikes returned by the LCMS were analysed for the area under the curve (AUC) as a measure of relative concentration.

*24 h profile:* Male C57BL/6 J mice were administered AC (100 mg kg^−1^ p.o., *N* = 4 per time point) suspended in vehicle containing 1% (w/v) hydroxyethylcellulose, 0.25% (v/v) polysorbate 80, and 0.05% (v/v) antifoam in Milli-Q water. At specific times (0.5–24 h), the mice were sacrificed and the brain rapidly dissected out and frozen. For HPLC analysis, the frozen tissue was allowed to thaw quickly and sonicated in 4 volumes (m/v) of acetonitrile: 10 mM ammonium formate, pH5 (95:5 v/v) using a Vibra-Cell sonic disruptor. Samples were left to stand on ice (4 °C) for 1 h, before centrifugation at 20,000 g for 15 min. The supernatant was removed and analysis of AC was carried out using HPLC coupled to electrochemical detection. 20 ul of each sample was injected (Triathlon, Spark Holland, Netherlands) and quantified against a calibration curve. Data was collected using Analyst 1.6.1 chromatography software (Sciex, Alderley Park, UK). A 4-parameter logistic fit was performed on all data prior to expression as ng g^−1^ wet weight tissue.

### Cell culture and quantification of receptor internalisation

#### tsA201 cell culture and transfection

TsA201 cells (ECACC catalogue no. 85120602) were maintained in growth media containing Dulbecco’s Modified Eagle Medium *(DMEM)*, 10% foetal calf serum, 1% non-essential amino acids, 1% penicillin (10,000 U ml^−1^), and streptomycin (10 mg ml^−1^) (all ThermoFisher, UK) in a humidified incubator at 37 °C/5% CO2 and then plated for transfection onto poly-L-lysine (0.1 mg ml^−1^; Sigma, UK) coated 13-mm glass coverslips. The cells were transfected with relevant cDNA using lipofectamine 3000 (Invitrogen, UK) and incubated at 37 °C/5% CO2 for 24–48 h before being imaged for receptor internalisation studies.

#### Receptor internalisation

TsA201 cells were washed with warmed serum-free DMEM 24–48-h post-transfection, following which 2-ml serum-free DMEM containing the relevant concentration of the drug of interest was added and the cells placed in the incubator for 45 min. Coverslips were then transferred to a HEPES-based buffer containing in mM: NaCl 140, KCl 2.5, MgCl_2_ 2, HEPES 10, D-glucose 10, and CaCl_2_ 2 with pH adjusted to 7.4 ± 0.02 and osmolarity corrected with sucrose to 310 ± 2 mOsm and confocal images obtained using a Leica SP5 confocal microscope. Images were analysed using ImageJ software with two regions of interest drawn, one for the whole cell and one for cytoplasm alone. The intensity of GFP fluorescence was quantified for each region and receptor internalisation calculated as GFP_cyto_/GFP_whole_. All data was acquired from cells with *n* = number of cells taken from at least 5 separate transfected tsA201 cultures.

### Behavioural testing

**Fig. 1:**
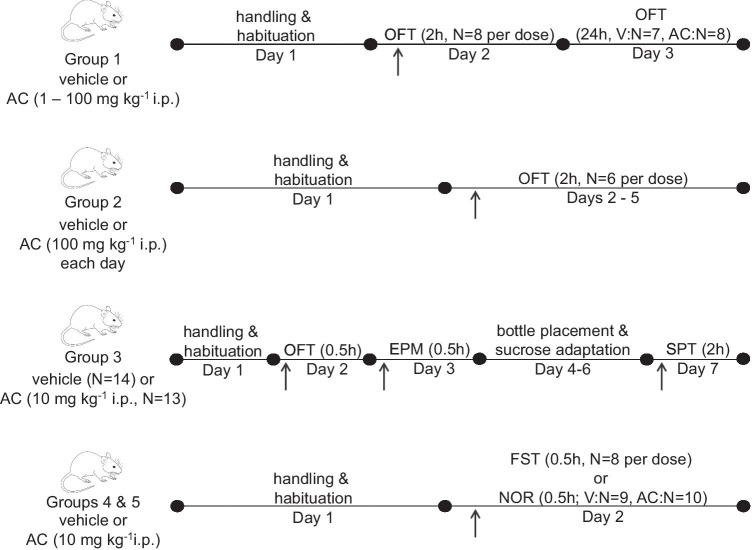
Schematic representation of the experimental protocols used to determine the effect of AC-264613 (i.p.) on mouse behaviour

For all behavioural testing, male mice (27.3 ± 0.4 g) were habituated to the relevant apparatus prior to experiments with drugs (AC and SLIGRL-NH_2:_ 1–100 mg kg^−1^) made up in a 0.9% saline solution including 1% Tween 80 and injected intraperitoneally (i.p.; maximum volume: 0.7 mL) as a suspension following warming and sonication. A schematic of the experimental timeline is shown in Fig. 1 with all apparatus thoroughly cleaned between sessions.

#### Open-field test (OFT)

OFT experiments were performed as previously described (Abulkassim et al. 2016; Pytka et al. 2020) in three groups of mice (Fig. 1): group 1, 2-h postinjection, *N* = 8 per dose; group 2, 2-h postinjection following repeated injections at the same time over 4 days, *N* = 6 per dose; group 3, 0.5-h postinjection, vehicle *N* = 14, AC (10 mg kg^−1^) *N* = 13.

#### Elevated plus maze (EPM)

The day after OFT in group 3, EPM was performed using mice (vehicle *N* = 14; AC (10 mg kg^−1^) *N* = 13) as previously described (Abulkassim et al. 2016; Abdul Rahman et al. 2016).

#### Sucrose preference test (SPT)

SPT was performed following OFT and EPM in group 3 (Fig. 1) as previously described (Pytka et al. 2020) with the amount of sucrose solution and water consumed was measured 2 h (vehicle *N* = 14; AC (10 mg kg^−1^) *N* = 13) and 24 h (AC (10 mg kg^−1^) *N* = 12) postinjection and the % sucrose intake and total volume drunk calculated.

#### Forced swim test (FST)

The FST was performed using group 4 (Fig. 1) based on previously described methods (Porsolt et al. 1977). Following handling and habituation on day 1, mice (*N* = 8 per treatment) were placed in a transparent glass vessel (25 cm in height, 14 cm in diameter) filled with 10 cm of water at 24 ± 2 °C on day 2 and activity recorded using Ethovision software. The activity was manually scored 0.5-h postinjection during the last 5 min of a single 6-min test session.

#### Novel object recognition (NOR)

The NOR test was performed using group 5 (vehicle *N* = 9; AC *N* = 10; Fig. 1) using the same apparatus and conditions as used for the OFT. On day 1, mice were handled and habituated for 15 min. For the sample phase on day 2, mice were initially allowed to explore two identical objects for 10 min, with the time spent exploring the two identical objects recorded using Ethovision software and scored by hand. Following injection with the relevant treatment, the choice phase was performed 0.5 h later, with the mouse allowed to explore two objects, one of which is novel, for 10 min. The time spent exploring the novel object compared to the familiar object was scored by hand and the discrimination ratio (time exploring novel object/total object exploration time × 100) calculated.

### Electrophysiology

Coronal brain Sects. (300 µm) containing the lateral habenula (LHb) were prepared from male and female C57BL/6 J mice (*N* = 8) using a Leica VT1200S vibratome (Leica Biosystems, UK) as previously described (Webster et al. 2020). Following sectioning, slices were incubated in oxygenated sucrose ACSF at 35 °C for 30 min and then incubated for a further 30 min at room temperature in ACSF containing (in mM) NaCl 115, NaHCO_3_ 25, KCl 3, NaH_2_PO_4_ 1.25, CaCl_2_ 2, MgCl_2_ 1, sodium pyruvate 3, and glucose 10. Visually guided whole-cell patch-clamp recordings were obtained from LHb neurons using glass micropipettes (5–8 MΩ) filled with an intracellular solution containing (in mM:) potassium gluconate 125, HEPES 10, KCl 6, EGTA 0.2, MgCl_2_ 2, Na-ATP 2, Na-GTP 0.5, and sodium phosphocreatine 5, with the pH adjusted to 7.2 with KOH, with data acquired using AxoGraph X software (www.axograph.com). Once in whole-cell configuration, spontaneous action potential (AP) firing was recorded in current clamp at the resting membrane potential with neurons with AP firing > 0.5 Hz considered to be spontaneously active, whereas neurons with AP firing < 0.5 Hz were deemed silent (Hu et al. 2020). Spontaneous excitatory postsynaptic currents (sEPSCs) were recorded in voltage clamp at − 70 mV following characterization of neuron type in current clamp. All data was analysed offline using AxoGraph X.

### Microglial isolation and culture

Microglia were isolated and cultured from C57BL/6 J male mice as described previously (Grabert and McColl 2018). Once isolated, microglia were resuspended in DMEM/F-12 (ThermoFisher Scientific, UK) supplemented with 1% PenStrep, 10% FBS, 500 pg/mL rhTGFβ-1 (Miltenyi Biotec, UK), and 10 pg/μL mCSF1 (RandD Systems, UK) and plated out at 40,000 cells/well onto a flat-bottom TC-treated 96-well plate (Corning, UK) coated with poly-L-lysine. Cells were cultured for 7 days at 37 °C in a humidified incubator under 5% CO_2_ with a half media change on day 3.

### Quantitative real-time PCR

C57BL/6 J male mice (10–12 weeks old) were given a single injection of either AC (100 mg kg^−1^) or vehicle and the cerebellum, hippocampus, and hypothalamus collected 2 h (*N* = 5 per group) or 24 h (*N* = 5 per group) postinjection and stored in RNAlater tissue storage solution (Life Technologies, UK) at < 4 °C until processing. Total RNA was isolated from the brain regions and reverse transcribed, with cycle threshold (C_t_) values generated as described previously (Abulkassim et al. 2016). qRT-PCR results were analysed using the relative quantification method of comparative Ct (ΔΔCt), with β-actin acting as the calibrator gene. All primer sequences and RT-PCR methods were as previously described (Abulkassim et al. 2016), with reactions run in triplicate.

### ELISA

#### Serum

C57BL/6 J male mice were euthanised by cervical dislocation at 2 and 24 h post-vehicle (*N* = 13) and AC (100 mg kg^−1^, 2 h N = 10, 24 h N = 10) injection and blood harvested immediately. Serum was isolated from whole blood samples and analysed for IL-1β (#88–7013-88), IL-6 (#88–7064-88), TNF-α (#88–7324-88), and IFN-γ (#88–7314-88) according to manufacturer’s instructions (ThermoFisher, UK).

#### Spleen and lymph nodes

C57BL/6 J male mice (N = 5) were euthanised as described above and spleens and lymph nodes harvested. Single-cell suspensions were cultured in 24-well plates at 2 × 10^6^ cells mL^−1^ and exposed to AC (50 µM) or vehicle controls. Supernatants were collected after 2 and 24 h, with IL-1β, IL-6, TNF-α, and IFN-γ levels determined using ELISA kits as described above.

#### Microglia

Microglia (7 days in culture) were exposed to either AC (50 µM), lipopolysaccharide (LPS; 100 ng mL^−1^), or vehicle control for 2 or 24 h. Following cell stimulation, supernatant was removed and analysed for IL-1β (#DY401-05), IL-6 (#DY406-05), and TNF-α (#DY410-05; all R&D Systems, UK).

### Data analysis and statistics

All data are expressed as mean ± S.E.M. and *N* = the number of animals used and *n* = number of LHb neuronal recordings with normality of all data sets determined with Shapiro–Wilk tests. For the electrophysiological experiments, no difference in AC effect was found between sexes, so data for both sexes was combined. Data were then compared, with tests used highlighted in the relevant figure legend, by unpaired or paired two-tailed student’s *t* tests, one-way analysis of variance with either a Dunnett’s or Bonferroni post hoc test, or two-way repeated-measures ANOVA with a Bonferroni post hoc test as appropriate using GraphPad Prism (v5.0 or v9.0) or Minitab (v19.0). Differences were considered significant when *P* < 0.05.

## Results

### AC crosses the BBB in a time-dependent manner

**Fig. 2:**
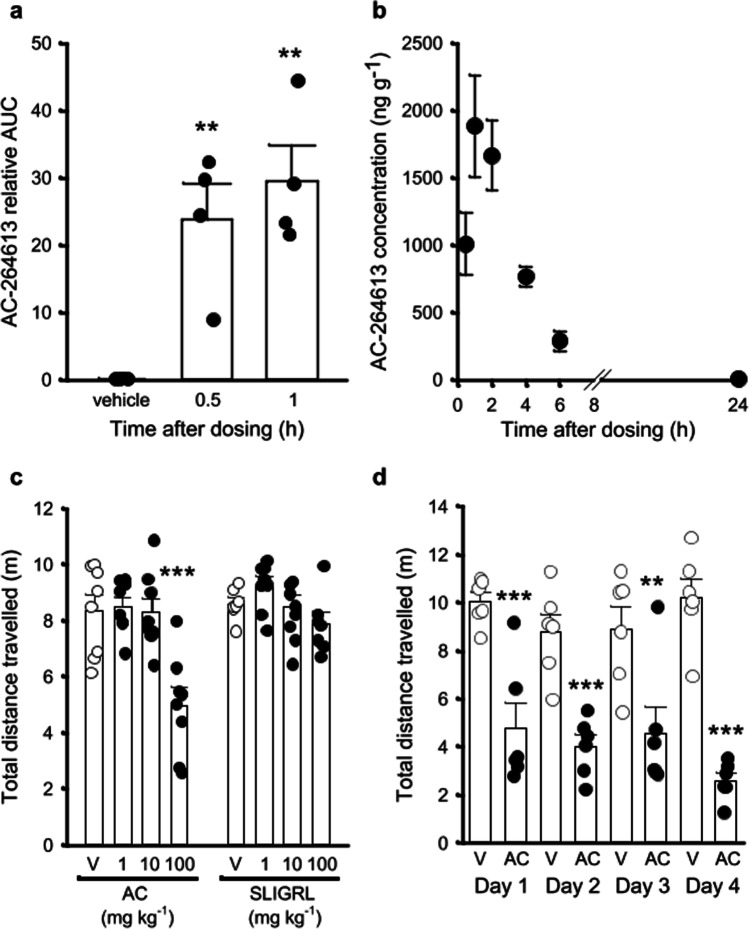
AC-264613 crosses the BBB following peripheral administration and dose-dependently reduces locomotor activity. **a** AC (10 mg kg^−1^ i.p., N = 4 per group) is present in brain tissue 0.5 and 1-h post-administration (** = *p* < 0.01. One way ANOVA with Dunnett’s post hoc test). **b** AC (100 mg kg^−1^ p.o., N = 4 per time point) peaks 1-h post-administration whereas only low levels were detected 24-h post-administration (one-way ANOVA with Dunnett’s post hoc test). **c** AC (100 mg kg^−1^ i.p.) reduces locomotor activity 2-h postinjection (*N* = 8 for both AC and vehicle, *** = *p* < 0.001 vs vehicle control, one-way ANOVA with Dunnett’s post hoc test) whereas SLIGRL-NH_2_ was without effect (*N* = 8 for all SLIGRL-NH_2_ doses, *N* = 7 for vehicle). **d** AC (100 mg kg^−1^ i.p.,) reduces locomotor activity following daily injections over a 4 day period (*N* = 6 for both AC and vehicle, ** = *p* < 0.01, *** = *p* < 0.001 both vs vehicle control, two-way repeated measures ANOVA with Bonferroni post hoc test)

Given that the consequence of PAR2 activation in mouse behaviour has been hindered due to the poor bioavailability of ligands, we initially examined whether PAR2 activators cross the BBB and enter the brain. In initial experiments, AC (10 mg kg^−1^ i.p., N = 4) was detected in brain samples when compared to the AC standard at 30 (*N* = 4, *p* = 0.006 v vehicle control) and 60 (*N* = 4, *p* = 0.002 v vehicle control) min postinjection, respectively (Fig. 2a). In contrast, SLIGRL-NH_2_ (10 mg kg^−1^ i.p., N = 4) was not detected in brain samples at both time points. Having established that AC enters the brain in mice post i.p. injection, we next investigated the pharmacokinetic profile of AC over a 24 h period when administered orally. Following a single dose of AC (100 mg kg^−1^ p.o.), AC was detected within the brain (*F*_(6,21)_ = 14.80, *N* = 4, *P* < 0.001) with levels peaking 1-h post-administration but was almost undetectable 24-h post-administration (Fig. 2b).

### AC impairs locomotor activity

Having established that AC crosses the BBB and enters the brain, we first confirmed AC’s PAR2 selectivity. AC induced PAR2-GFP internalisation (see Online Resource 2) but was without the effect of PARs 1 and 4 or P2Y12, thus confirming its PAR2 selectivity as previously reported (Gardell et al. 2008). We next examined the consequence of PAR2 activation on mouse behaviour. Following habituation, mice were randomly assigned to treatment groups and the effect on locomotor activity assessed 2-h and 24-h post-injection. AC (1–100 mg kg^−1^ i.p.) significantly impaired locomotor activity in male mice (*F*_(3,28)_ = 10.91, *P* < 0.001) with AC (100 mg kg^−1^ i.p., *N* = 8, *P* < 0.001, Fig. 2c) significantly impairing locomotor activity 2-h post-injection when compared to vehicle-injected controls, whereas lower concentrations were without effect (Fig. 2c). In contrast to AC, SLIGRL-NH_2_ did not alter locomotor activity at any dose investigated (1–100 mg kg^−1^, *N* = 8 for SLIGRL-NH_2_, *N* = 7 for vehicle, *F*_(3,27)_ = 2.07, *p* = 0.13, Fig. 2c). To determine whether AC (100 mg kg^−1^ i.p) impaired locomotor activity persisted, we next examined locomotor activity 24-h post-injection. However, no impairment in locomotor activity (*N* = 8, *p* = 0.955) was observed in male mice 24-h post-injection. Furthermore, daily administration of AC (100 mg kg^−1^ i.p., 4 consecutive days, *N* = 6) resulted in impaired locomotor activity in male mice 2-h post-injection compared to vehicle controls (*F*_(1,30)_ = 72.15, *p* < 0.001), but this was not significantly different across days (*F*_(3,30)_ = 2.04, *p* = 0.129, Fig. 2d).

### AC reduces sucrose preference but does not alter anxiety-related behaviour

**Fig. 3:**
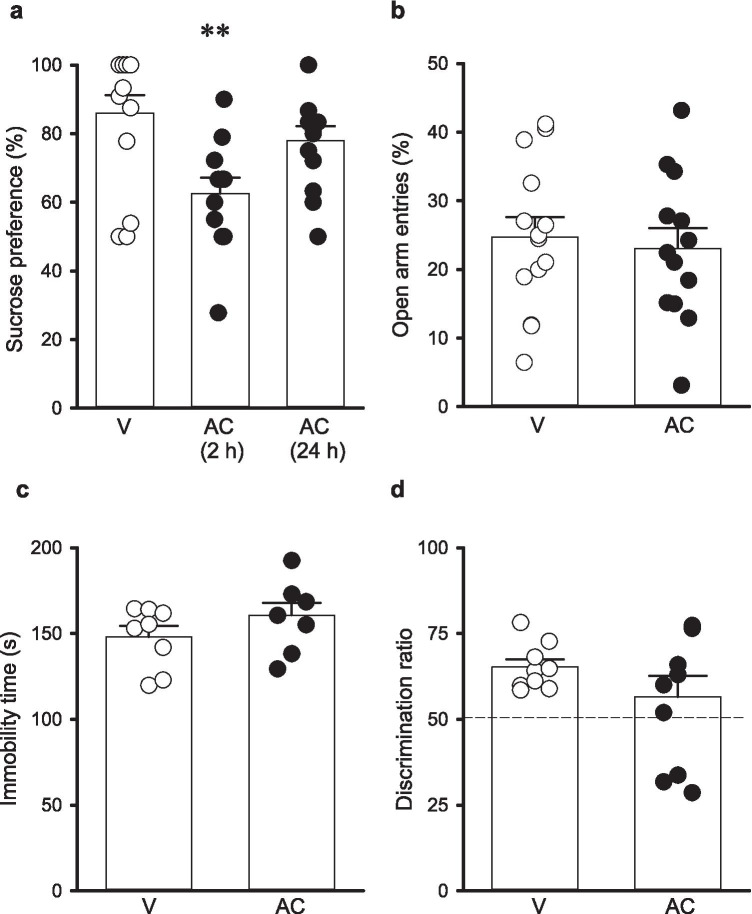
AC-264613 reduces sucrose preference but does not affect anxiety-like behaviour or recognition memory. **a** AC (10 mg kg^−1^ i.p.) reduces sucrose preference 2-h postinjection (*N* = 13 for AC, *N* = 14 for vehicle, ** = *p* < 0.01 vs vehicle control, one-way ANOVA with Dunnett’s post hoc test) but was without effect 24-h post-injection (*N* = 12). **b** AC (10 mg kg^−1^ i.p., *N* = 14, *P* = 0.693 vs vehicle control, unpaired student’s *t* test) did not alter open arm entries in the elevated plus maze compared to vehicle control (*N* = 13). **c** AC (10 mg kg^−1^ i.p., *N* = 8, *P* = 0.199 vs vehicle control, unpaired two-tailed student’s *t* test) did not alter immobility time in the forced swim test compared to vehicle control (*N* = 8). **d** AC (10 mg kg^−1^ i.p., *N* = 10, *P* = 0.220 vs vehicle control, unpaired two-tailed student’s *t* test) does not affect memory compared to vehicle control (*N* = 9) when examined in the novel object recognition test

We next examined whether PAR2 activation induces anxiety-like behaviour and/or anhedonia, and given the reduced locomotor activity observed with AC (100 mg kg^−1^) and its pharmacokinetic profile, we investigated whether AC (10 mg kg^−1^ i.p.) modulated anxiety-like behaviour and sucrose preference 0.5-h postinjection. In agreement with lower concentrations of AC not altering locomotor activity 2-h postinjection, AC (10 mg kg^−1^) did not affect locomotor activity 0.5-h postinjection (*N* = 13, *P* = 0.961). In addition, no changes were observed in the percentage time spent in the centre square during the OFT for both the higher and lower doses of AC (100 mg kg^−1^ 2-h postinjection: *N* = 8, *P* = 0.640; 10-mg kg^−1^ 0.5-h postinjection: *N* = 13, *P* = 0.942). We also examined whether AC altered performance in the EPM. Similar to our observation in the OFT regarding time spent in the centre square, AC (10 mg kg^−1^ i.p.) did not alter the percentage open arm entries in the EPM 0.5-h postinjection (*N* = 13, *P* = 0.693, Fig. 3b). However, in contrast to the lack of effect on locomotor activity and anxiety-like behaviour, AC (10 mg kg^−1^ i.p.) significantly reduced sucrose preference (*F*_(2,37)_ = 6.06, *p* = 0.006) with AC (10 mg kg^−1^ i.p.) reducing sucrose preference 0.5-h postinjection (*N* = 13, *p* = 0.003, Fig. 3a) compared vehicle controls (*N* = 14). However, no change in sucrose preference was observed 24-h postinjection (*N* = 12, *P* = 0.401, Fig. 3a), and no change was observed in the total volume of liquid drunk following AC (10 mg kg^−1^ i.p.) 2-h (*N* = 13, *P* = 0.310) or 24-h (*N* = 13, *P* = 0.343) postinjection.

### AC does not alter performance in FST and NOR tasks

Having established that AC (10 mg kg^−1^ i.p.) induced anhedonia, a symptom associated with depression-like behaviour, we also investigated whether AC (10 mg kg^−1^ i.p.) increased immobility in the FST. However, AC (10 mg kg^−1^ i.p.) did not alter immobility time when examined 0.5-h postinjection (*N* = 8, *P* = 0.199, Fig. 3c). Finally, we investigated whether AC altered performance in the NOR test. However, AC (10 mg kg^−1^ i.p.) did not alter the discrimination ratio when investigated 0.5-h postinjection (*N* = 10, *P* = 0.220, Fig. 3d).

### AC reduces spontaneous action potential firing within the lateral habenula

**Fig. 4:**
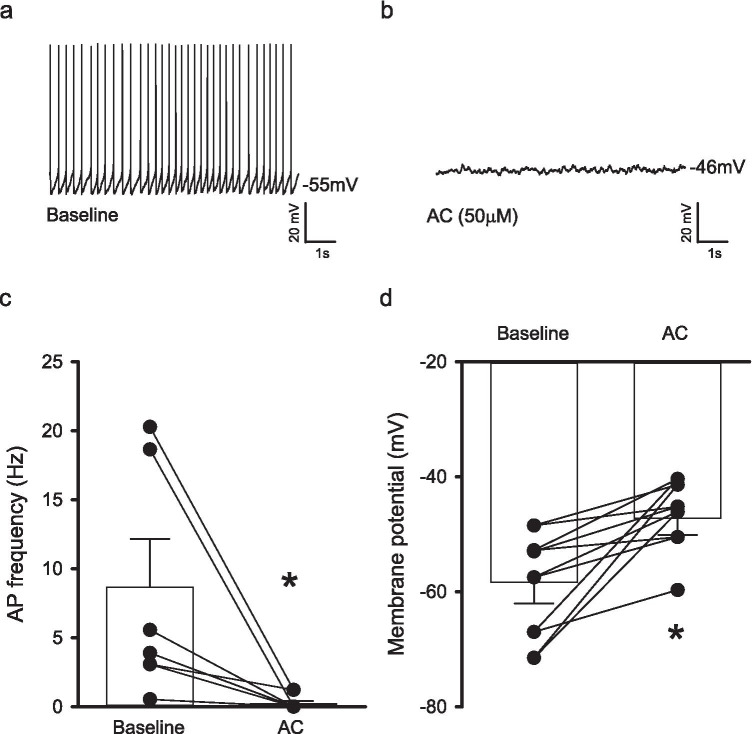
AC-264613 impairs action potential firing and induces depolarisation in spontaneously firing lateral habenula neurons. **a + b** Representative traces showing action potential firing and neuronal membrane potential in the absence and presence of AC (50 µM). **c** AC (50 µM, *n* = 6 from 5 mice) reduces the action potential frequency in spontaneously firing lateral habenula neurons (* = *p* < 0.05, two-tailed paired student’s *t* test). **d** AC (50 µM, *n* = 6 from 5 mice) depolarises spontaneously firing lateral habenula neurons (* = *p* < 0.05, two-tailed paired student’s *t* test)

Several brain regions have been proposed to play a role in depression-like behaviour, with intense interest recently focusing on the role of the lateral habenula (LHb; Browne et al. 2018; Hu et al. 2020). Hence, we examined whether AC modulated the passive and active properties of spontaneously firing LHb neurons. Application of AC (50 µM) significantly reduced AP firing (*n* = 6 from 5 mice, *p* = 0.03, Fig. 4a, b) whereas DMSO (0.1% v/v) as a vehicle control was without effect (*n* = 4 from 3 mice, *p* = 0.22). In addition, AC (50 µM) selectively depolarised spontaneously firing LHb neurons (*n* = 6 from 5 mice, *p* = 0.03, Fig. 4a, c). However, AC (50 µM) did not alter sEPSC frequency (*n* = 6 from 5 mice, *p* = 0.82) or amplitude (*n* = 6 from 5 mice, *p* = 0.18) recorded from all LHb neurons. In addition, D-AP5 (50 µM) did not impair AC-induced reductions in AP firing (*n* = 5 from 5 mice, *p* = 0.08) but impaired AC-induced depolarizations (*n* = 5 from 5 mice, *p* = 0.03) observed in spontaneously active LHb neurons. In contrast, AC (50 µM) did not induce AP firing (*n* = 7 from 5 mice, *p* = 0.88) or alter the resting membrane potential (*n* = 7 from 5 mice, *p* = 0.21) in silent LHb neurons.

### AC induces differential serum and brain cytokine profiles

**Fig. 5:**
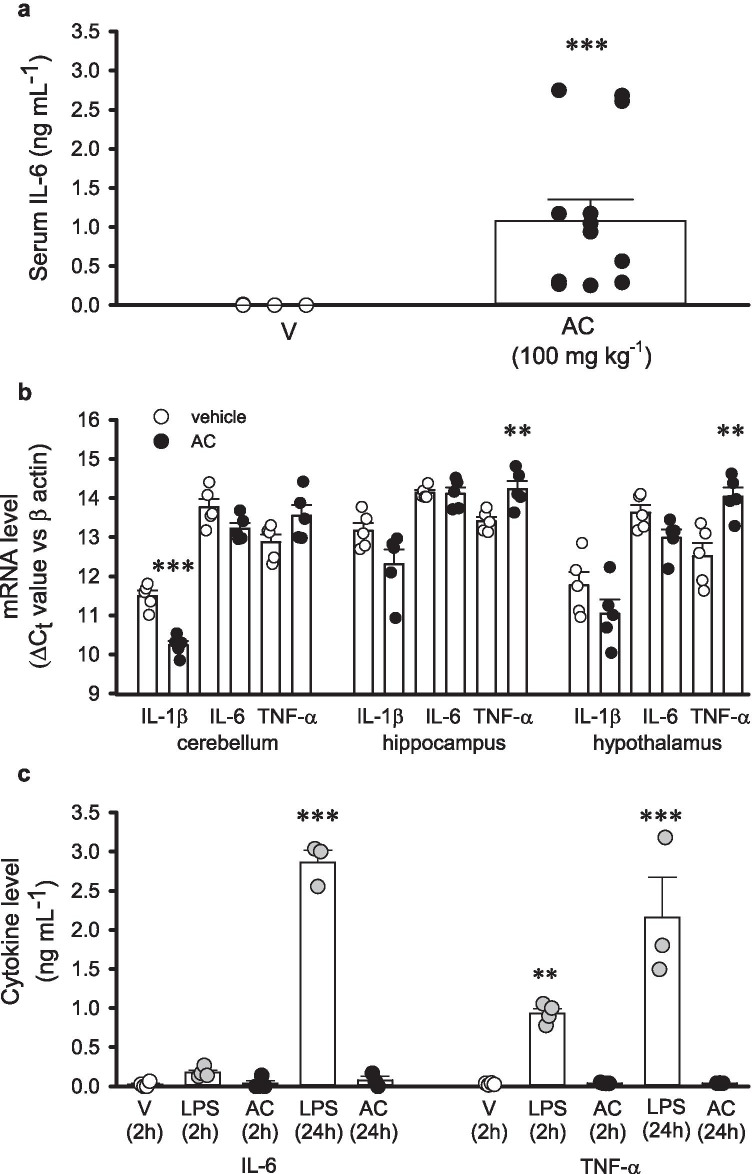
AC-264613 alters peripheral and central cytokine expression. **a** AC (100 mg kg^−1^ i.p., *N* = 10) selectively elevates blood serum IL-6 levels 2-h postinjection with IL-1 β, TNF-α, or IFN-γ being undetectable (*** = p < 0.001 vs vehicle control, unpaired two-tailed student’s *t* test). **b** IL-1β mRNA levels are increased in the cerebellum, as shown by reduced cycle threshold for detection (ΔC_t_ v β actin), 2-h post AC-injection (100 mg kg^−1^ i.p., *N* = 5) whereas TNF-α mRNA levels are decreased 2-h post-injection in the hippocampus and hypothalamus (** = *p* < 0.01, *** = *p* < 0.001 both vs vehicle control, one-way ANOVA with Dunnett’s post hoc test). **c** LPS (100 ng mL^−1^, N ≥ 3) but not AC-264613 (50 µM, N ≥ 3) induces IL-6 and TNF-α release from cultured primary microglia (** = *p* < 0.01, *** = *p* < 0.001 both vs vehicle control, one-way ANOVA with Dunnett’s post hoc test)

Having established that AC impairs locomotor activity and induces anhedonia and given the established link between depression and inflammation (Miller and Raison 2016; Köhler et al. 2017; Pape et al. 2019), we next investigated whether AC altered blood and/or brain cytokine profiles. AC (100 mg kg^−1^ i.p.) significantly increased serum levels of IL-6 2-h post-injection (*N* = 10, *P* < 0.001, Fig. 5a) compared to vehicle controls (*N* = 13) when investigated using ELISA, whereas 24-h postinjection, IL-6 was below detectable levels for both AC and vehicle control. In contrast, serum IL-1β, TNF-α, or IFN-γ were below detectable levels for both AC (100 mg kg^−1^ i.p., *N* = 10) and vehicle control (*N* = 13) at both time points. We also examined whether the brain cytokine profile was altered following AC (100 mg kg^−1^ i.p.). In contrast to serum, IL-6 mRNA levels were not significantly changed 2-h postinjection in the cerebellum (*N* = 5, *P* = 0.07, Fig. 5b), hippocampus (*N* = 5, *P* = 0.87, Fig. 5b), or hypothalamus (*N* = 5, *P* = 0.05, Fig. 5b). However, IL-1β mRNA levels were significantly increased in the cerebellum (*N* = 5, *P* < 0.001, Fig. 5b) whereas mRNA levels were unaltered in the hippocampus (*N* = 5, *P* = 0.08, Fig. 5b) and hypothalamus (*N* = 5, *P* = 0.19, Fig. 5b). Strikingly, TNF-α mRNA levels were significantly reduced in the hippocampus (*N* = 5, *P* = 0.009, Fig. 5b) and the hypothalamus (*N* = 5, *P* = 0.005 v vehicle controls, Fig. 5b) whereas no changes were observed in the cerebellum (*N* = 5, *P* = 0.08, Fig. 5b). With regard to IFN-γ mRNA levels, no changes were observed in the cerebellum (*N* = 5, *P* = 0.418), hippocampus (*N* = 5, *P* = 0.492), or hypothalamus (*N* = 5, *P* = 0.104).

### AC does not induce cytokine release from innate and adaptive immune cells

Given that AC-induced behavioural changes may be mediated, at least in part, by changes in the cytokine profile, we next examined their potential source. Stimulation of cultured primary microglia with AC (50 µM) did not induce IL-6 or TNF-α release at both 2-h (*N* = 4) and 24-h (*N* = 3) time points (Fig. 5c), whereas LPS (100 ng mL^−1^) induced a significant increase in IL-6 following a 24 h exposure (N = 3, p < 0.001, Fig. 5c) but no significant release was detected following LPS (100 ng mL^−1^) for 2 h (*N* = 4). Similarly, LPS (100 ng mL^−1^) resulted in significant increases in TNF-α following both 2 h (*N* = 4, *p* = 0.012, Fig. 5c) and 24 h (*N* = 3, *p* < 0.001, Fig. 5c) treatments. Finally, no changes in IL-1β levels were detected at either time point for both AC (50 µM) and LPS (100 ng mL^−1^). In addition, IL-1β, IL-6, and TNF-α levels were undetectable in either control lymphocyte cultures (*N* = 5, 2, and 24 h) or those treated with AC (50 µM, *N* = 5, 2, and 24 h).

## Discussion

In the present study, we reveal that the PAR2 activator, AC, crosses the BBB, impairs locomotor activity, and induces anhedonia. These AC-induced behavioural changes are associated with the selective increase in blood IL-6 levels as well as modulating cytokine mRNA expression in selective brain regions.

We have previously shown that LPS-induced sickness behaviour is impaired in PAR2^−/−^ mice (Abulkassim et al. 2016), but whether PAR2 activation induces behavioural changes similar to those observed in sickness and depression-like behaviour is unknown. This is primarily due to the proteolytic degradation of peptide and peptide mimetics used as PAR2 research tools, particularly when used in vivo (Ramachandran et al. 2012; McIntosh et al. 2020). However, the development of novel PAR2 small molecule activators including AC-264613 (Gardell et al. 2008) raised the possibility that these molecules cross the BBB and therefore can be utilized to investigate the consequence of PAR2 activation in behavioural tests. We initially confirmed that AC does indeed cross the mouse BBB and enter the brain when injected i.p. whereas SLIGRL-NH_2_ was not detected. Our finding regarding SLIGRL-NH_2_ is in contrast to a previous study that showed the presence of SLIGRL-NH2 in the brain when examined in Genetic Absence Epilepsy Rats from Strasbourg (GAERS; Lohman et al. 2009). However, whether these discrepancies are due to species differences or BBB dysfunction in the GAERS rats is unknown. Nonetheless, we then proceeded to examine the pharmacokinetic profile of AC, which revealed that it peaked within the brain 1-h post-administration but was undetectable 24-h post-administration, a similar PK profile to that previously observed within blood plasma (Gardell et al. 2008). We next tested AC’s selectivity for PAR2 in vitro using internalisation assays, with our findings confirming its PAR2 selectivity as reported previously (Gardell et al. 2008), However, in the absence of antagonist data or experiments using PAR2^−/−^ mice, whether AC retains this PAR2 selectivity in vivo remains to be elucidated. Having established that AC crosses the BBB and is PAR2 selective in vitro, we examined the consequence of PAR2 activation on mouse behaviour. Having previously shown that LPS-induced changes in locomotor activity and sucrose preference are altered in sickness behaviour when investigated in PAR2^−/−^ mice (Abulkassim et al. 2016), we initially examined whether administering AC would induce changes in these behavioural assays in a similar manner. Indeed, AC administration reduced locomotor activity and sucrose preference, which is consistent with a role for PAR2 in sickness behaviour. We also reveal that no long-term effects were present as locomotor activity was unaltered when examined 24-h postinjection and repeated injections resulted in a similar reduction in activity on each day of injection. This confirms that AC-induced behavioural changes occur in a time-dependent manner consistent with its pharmacokinetic profile and that AC does not induce either behavioural tolerance or sustained behavioural changes. However, as PAR2 is quiescent under physiological conditions with several behavioural assays being unaltered in PAR2^−/−^ mice (Abulkassim et al. 2016), our data suggests that PAR2, although present within the CNS (D’Andrea et al. 1998; Striggow et al. 2001; Riek-Burchardt et al. 2002; Jin et al. 2005; Noorbakhsh et al. 2006; Bushell et al. 2016), is only activated and contributes to behavioural changes under certain conditions. Nevertheless, the fact that AC administration results in reduced locomotor activity and sucrose preference is striking in its similarity to changes observed in both sickness and depression-like behaviour. As such, we then examined whether AC modulates behaviour in the FST. No changes in immobility time were observed indicating that PAR2 activation does not modulate this behaviour. However, despite the FST being used extensively to examine anti-depressant activity, there is some debate as to whether it is a measure of depression-like behaviour or more related to anxiety-like behaviour (Gururajan et al. 2019; Molendijk and de Kloet 2021). Given that AC does not alter anxiety-like behaviour in the OFT and in the EPM, our findings in the FST indicate that PAR2 activation does not affect anxiety-like behaviour. In addition, AC did not alter performance in the NOR memory task despite our previous study revealing that PAR2 activation resulted in the long-term depression of hippocampal synaptic transmission (Gan et al. 2011) and the established link between hippocampal synaptic plasticity and memory (Bliss and Collingridge 1993; Aggleton and Morris 2018). However, our behavioural data indicates that AC administration elicits behavioural changes in male mice that are similar to those seen in sickness and depression-like behaviour. Nevertheless, we are cognisant that MDD is more prevalent in women and that sex differences occur in rodent models of depression (Seney and Sibille 2014; Franceschini and Fattore 2021). Hence, it would be interesting in future studies to examine whether there are sex related differences in AC-induced behavioural changes.

Given our findings, we then sought to determine the underlying causes of these behavioural changes. There has been intense interest recently regarding the role of the LHb in depression and depression-like behaviour, with increased neuronal activity being evident in a variety of models of depression-like behaviour (Mirrione et al. 2014; Lecca et al. 2016; Tchenio et al. 2017; Yang et al. 2018). Indeed, several antidepressant treatments have been shown to modulate habenula function and relieve depression in patients (Sartorius et al. 2010; Vitkauskas and Mathuru 2020; Wang et al. 2021). Hence, we sought to determine whether PAR2 activation modulates LHb neuronal activity and thus examine whether the LHb is involved in the AC-induced behavioural changes. However, application of AC resulted in the strong inhibition of AP firing in spontaneously active LHb neurons, despite also inducing depolarization of the membrane potential but was without effect on silent LHb neurons. Although our findings do not agree with the conventional correlation between depression-like behaviour and increased LHb neuronal activity, we nonetheless show that AC selectively modulates the activity of spontaneously active LHb neurons, which are believed to be more strongly associated with depressive symptoms, and thus this does not exclude that the AC-induced depression-like behaviour is mediated via the LHb. Whilst we focused on the LHb in the present study, other brain regions are also implicated to have key roles in depression-like behaviour including the hippocampus and the prefrontal cortex (Malykhin and Coupland 2015; Boku et al. 2018; Yan and Rein 2021). The data presented here agrees with our previous study examining hippocampal activity (Gan et al. 2011), with PAR2 activation reducing neuronal activity in both brain regions but how these ex vivo findings relate to depression-like behaviour remains to be elucidated as is the consequence of PAR2 activation on neuronal firing in other brain regions associated with depression-like behaviour.

It is now well established that inflammatory mediators are elevated in both patients with depression and rodent models of depression-like behaviour (Miller and Raison 2016; Dantzer 2017; Felger 2019; Branchi et al. 2020). Similarly, PAR2 has been linked to inflammatory diseases including rheumatoid arthritis and inflammatory bowel disease (Hollenberg et al. 2014; McCulloch et al. 2018; Chandrabalan and Ramachandran 2021). In agreement with the link between PAR2 and inflammation, we found that AC (100 mg kg^−1^) led to elevated IL-6 levels in blood serum 2-h postinjection whilst IL-1β mRNA was increased and TNF-α mRNA levels reduced in the brain. Whilst cytokine levels were not examined 0.5-h postinjection, a time point where sucrose preference was reduced, the link between PAR2 and inflammation and inflammation and anhedonia suggest that cytokines are likely to be involved. A link between elevated blood IL-6 levels and depression is well established in both patients (Dowlati et al. 2010; Haapakoski et al. 2015; Nobis et al. 2020) and rodent models (Krishna et al. 2016; Rodrigues et al. 2018; Wickens et al. 2018) with IL-6^−/−^ mice being resistant to induced depression-like behaviour (Chourbaji et al. 2006; Monje et al. 2011). Strikingly, IL-6 was the only cytokine whose serum levels were elevated following AC injection, with other cytokines including IL-1β and TNF-α being below detectable levels. Given that all three cytokines were undetectable in AC-stimulated immune cell preparations from lymph nodes and spleens, this indicates that T and B cells are unlikely to be the source of IL-6. Although PAR2-induced release of IL-1β and IL-6 was previously reported in human peripheral blood monocytes (Johansson et al. 2005), it is unknown whether this is also true of mouse monocytes. Hence in future studies, it would be interesting to undertake a more extensive cytokine and chemokine array in distinct immune cell populations to determine whether other mediators are involved, identify their source and further probe the link between peripheral cytokine levels and centrally mediated depression-like behaviour.

In contrast to IL-6 levels in the blood, brain IL-6 mRNA levels were not significantly altered although a trend towards increased expression was observed in the cerebellum and hypothalamus. However, significantly increased IL-1β mRNA levels were observed in certain brain regions whereas in contrast, TNF-α mRNA levels were reduced. Increased brain cytokine levels are regularly reported in rodent models of sickness and depression-like behaviour (Dantzer 2017; Takahashi et al. 2018); therefore, the observed increases in IL-1β mRNA following AC injection are consistent with it playing a role in the PAR2-induced behavioural changes and with elevated cytokine levels reported for other PAR2-related inflammatory disorders (McCulloch et al. 2018; Chandrabalan and Ramachandran 2021). Furthermore, we have previously shown that brain IL-1β levels are elevated in LPS-induced sickness behaviour, a condition ameliorated in PAR2^−/−^ mice, which further supports our conclusion that PAR2 activation results in altered cytokine expression, resulting in AC-induced behavioural changes. Recently, microglia have been proposed to play a central role in depression-like behaviour and are proposed as a key cell type involved in the associated elevated cytokine levels (Frank et al. 2019; Deng et al. 2020). However, AC did not induce IL-6 or TNF-α release from primary mouse microglia, which is in contrast to previous studies that have reported elevated IL-6 or TNF-α following microglial PAR2 activation (Chen et al. 2012; Zhang et al. 2016). Hence, these data suggest that AC injection results in increased brain cytokine expression but PAR2 activation of microglia appears not to be involved. An alternative source for the cytokine changes may be via astrocytic activation. IL-1β and IL-6 are released from astrocytes (Araki et al. 2021) and recent studies using human postmortem tissue propose a role for astrocytes in MDD (O’Leary and Mechawar 2021; Zhang et al. 2021). Hence, astrocytes may play a role in the AC-induced behavioural changes, and this will be interesting to examine in future studies.

In conclusion, we show for the first time that PAR2 activation using a BBB permeable activator results in behavioural changes similar to those observed in sickness and depression-like behaviour. Changes in blood and brain cytokine expression are similar to those observed in both rodent models of sickness and depression-like behaviour and in patients with MDD, strongly indicating that PAR2-induced inflammatory mediators underlie this. Our findings, aligned with our previous study highlighting the role of PAR2 in LPS-induced sickness behaviour, suggest that PAR2 activation may be a novel model for examining behavioural changes associated with sickness and depression-like behaviour and that its inhibition may ameliorate these changes. Given the recent development of novel small molecule PAR2 antagonists (Cheng et al. 2017; Kennedy et al. 2018), these will allow further characterisation of the role that PAR2 plays in CNS disorders, which in turn may highlight PAR2 as a target for the development of novel antidepressants.

## Supplementary Information

Below is the link to the electronic supplementary material.

Supplementary file1 (PDF 223 KB)

Supplementary file2 (PDF 48 KB)
