# Supplementary material for: Protease-activated receptor 2 activation induces behavioural changes associated with depression-like behaviour through microglial-independent modulation of inflammatory cytokines
Source: Psychopharmacology (Berl). 2021 Dec 9;239(1):229–42. doi: 10.1007/s00213-021-06040-1 (PMC8770450; doi:10.1007/s00213-021-06040-1)
Supplement: Supplementary file 1 — Supplementary file1 (PDF 223 KB) [file 213_2021_6040_MOESM1_ESM.pdf]

Protease-activated receptor 2 activation induces depression-like behaviour through microglial-independent modulation of inflammatory cytokines.

Serge Moudio, Ashleigh Willis, Karolina Pytka, Roua Abulkassim, Ros R. Brett, Jack F. Webster, Christian Wozny, Mark Barbour, Hui-Rong Jiang, David G Watson, Josie C. van Kralingen, Scott M. MacKenzie, Michael Daniels, Barry W McColl, Sandra Sossick, Hugh N Nuthall & Trevor J. Bushell.

ESM1 Supplementary Methods

Pharmacokinetic profiling of AC

Initial LC-MS: Male C57BL/6J mice injected intraperitoneally (i.p.) with vehicle (N=4), AC (N=8, 10 mg kg^{-1}) or SLIGRL-NH_2 (N=8, 10 mg kg^{-1}) were killed by cervical dislocation at specific time points (0.5 - 1h) post-injection. The brains were rapidly removed and immediately frozen by immersion in -40 ˚C isopentane (2-methyl butane; Fisher, Scientific UK) and stored at -80 ˚C until required. For liquid chromatography-mass spectrometry (LC-MS), brain tissue (100 mg) was weighed and mixed with 200µl of 0.1% formic acid and 800µl of methanol using an Ultra-Turrax homogenizer. The mixture was transferred to a microcentrifuge tube and centrifuged for 10 min at 5000 RPM at room temperature (20 °C). The supernatant was removed and samples were then loaded onto an Orbitrap exactive mass spectrometer (ThermoFisher, UK) coupled to a Dionex HPLC system (Dionex, Camberley, UK). The gas flow in the ESI source was adjusted to sheath gas 60 (arbitrary units) and auxiliary gas 25 (arbitrary units) to give a flow rate was 0.3 ml/min. The capillary temperature was 275° C and the needle voltage was 4.5 kV. The instrument was calibrated according to the manufacturer's instructions prior to use and was internally calibrated by lock masses (positive ion mode m/z 83.06037 due to an acetonitrile dimer and negative ion mode 91.00368 due to a formic acid dimer). The elution 'finger-print' of AC (MW 400.1, elution time ≈16 min) and SLIGRL-NH_2 (MW 658, elution time ≈16.5 min) was initially determined using a known concentration with the column flushed with acetonitrile following each sample to remove any residue. Original signal spikes returned by the LCMS were analysed for area under the curve (AUC) as a measure of relative concentration.

24h profile: Male C57BL/6J mice were administered AC (100 mg kg^{-1} p.o., N=4 per time point) suspended in vehicle containing 1% (w/v) hydroxyethylcellulose, 0.25% (v/v) polysorbate 80 and 0.05%

(v/v) antifoam in milliQ water and maintained with free access to food and water. At specific times following the administration (0.5 – 24h), the mice were sacrificed and the brain rapidly dissected out and the brainstem and cerebellum removed. The brain was then weighed and frozen pending HPLC analysis. The frozen tissue was allowed to thaw quickly and sonicated in 4 volumes (m/v) of acetonitrile:10 mM ammonium formate, pH5 (95:5 v/v) using a Vibra-cell sonic disruptor set on 50% amplitude and pulsed for approximately 20 secs. Samples were left to stand on ice (4°C) for 1 hour, before centrifugation at 20,000 g for 15 min. The supernatant was removed and analysis of AC was carried out using HPLC coupled to electrochemical detection. 20ul of each sample was injected (Triathlon, Spark Holland, Netherlands) and quantified against a calibration curve. Data was collected using Analyst 1.6.1 chromatography software (Sciex, Alderley Park, UK). A 4-parameter logistic fit was performed on all data prior to expression as ng g^{-1} wet weight tissue.

Cell culture and quantification of receptor internalisation.

tsA201 cell culture and transfection: TsA201 cells (ECACC catalogue no. 85120602) were maintained in growth media containing Dulbecco's Modified Eagle Medium (DMEM), 10% foetal calf serum, 1% non-essential amino acids, 1% penicillin (10,000 U ml^{-1}) and streptomycin (10 mg ml^{-1}) (all ThermoFisher, UK) in a humidified incubator at 37 °C / 5% CO2. When the cells were at least 90% confluent, they were split and plated for transfection onto poly-L-lysine (0.1 mg ml^{-1}; Sigma, UK) coated 13mm glass coverslips. The cells were flooded after 2 h with 2 ml serum-free DMEM and transfected with either PAR1-GFP, PAR2-GFP, PAR4-GFP or hP2Y12-GFP cDNA (1 µg per transfection, all cDNAs were contained in a pcDNA 3.1 vector; kindly prepared by Dr Rothwell Tate, University of Strathclyde) using Lipofectamine 3000 (Invitrogen, UK). The transfection media was removed after 5h and replaced with growth media, with cells then incubated at 37 °C / 5% CO2 for 24-48 h before being imaged for receptor internalisation studies.

Receptor internalisation: TsA201 cells were washed with warmed serum free DMEM 24-48h post-transfection, following which 2 ml serum free DMEM containing the relevant concentration of the drug of interest was added and the cells placed in the incubator for 45 min. Coverslips were then transferred to a HEPES-based buffer containing in mM: NaCl 140, KCl 2.5, $MgCl_2$ 2, HEPES 10, D-glucose 10,

$CaCl_2$ 2 with pH adjusted to 7.4 ± 0.02 and osmolarity corrected with sucrose to 310 ± 2 mOsm and confocal images obtained using a Leica SP5 confocal microscope. Images were analysed using ImageJ software (Schneider et al., 2012) with two regions of interest drawn, one for the whole cell and one for cytoplasm alone. The intensity of GFP fluorescence was quantified for each region and receptor internalisation calculated as GFP_{cyto} / GFP_{whole}. All data was acquired from cells with n = number of cells taken from at least 5 separate transfected tsA201 cultures.

Behavioural testing

For all behavioural testing, male mice (27.3 ± 0.4g) were habituated to the relevant apparatus prior to experiments with drugs (AC & SLIGRL-NH_2: 1 – 100 mg kg^{-1}) made up in a 0.9% saline solution including 1% Tween 80 and injected intraperitoneally (i.p.; maximum volume: 0.7 mL) as a suspension following warming and sonication. A schematic of the experimental timeline is shown in Fig. 1 with all apparatus thoroughly cleaned with 10% Decon™ Decon 90 (ThermoFisher, UK) between sessions.

Open field test (OFT): OFT experiments were performed in three groups of mice (Fig.1): Group 1 - 2h post-injection, N=8 per dose; Group 2 – 2h post-injection following repeated injections at the same time over 4 days, N=6 per dose; Group 3 - 0.5h post-injection, vehicle N=14, AC (10mg kg^{-1}) N=13. In all cases, following handling and habituation, mice were placed in the centre of an open field box (40 x 40 x 40cm) made from black infrared light (IR)-translucent Perspex placed on an in IR light box (Tracksys, Nottingham, UK) as previously described [16, 24]. Total distance moved and entries into and time spent in a 14 x 14 cm centre square were recorded for 10 min by tracking software (Ethovision, Noldus, Netherlands) at the stated time post-injection.

Elevated plus maze (EPM): The day after OFT in group 3, mice (vehicle N=14; AC (10mg kg^{-1}) N=13) were placed in the centre of a plus-shaped maze with two open (30 x 5cm) and two closed arms (30 x 5cm, 15 cm walls) made of IR-translucent Perspex with integral IR light sources elevated 70cm from the floor (Tracksys, Nottingham, UK) as previously described [16, 25]. Entries into each type of arm were recorded for 10 min 0.5h post-injection by Ethovision software and the total number of entries and % open arm entries calculated.

Sucrose preference test (SPT): SPT was performed following OFT and EPM in group 3 (Fig. 1) as previously described [24]. Before the test, each mouse was singly housed in an MB1 cage (45 x 28 x 13 cm). Mice were presented with two identical bottles of water and the amount of water consumed for

each was measured for 24 hours in order to determine the preferred bottle position. For the next two days, mice were adapted to sucrose by replacing the water bottle in the non-preferred position with an identical bottle containing 1 % (w/v) sucrose solution. The 1% sucrose solution was freshly prepared by dissolving sucrose (Fisher, Scientific UK) in tap water, with the amount of water and sucrose consumed measured daily. Following the adaptation period (3 days), the sucrose preference test was performed with the position of the two bottles (water and 1% sucrose solution) randomly determined to avoid a place preference. The amount of sucrose solution and water consumed was measured 2h (vehicle N=14; AC (10mg kg^{-1}) N=13) and 24 hours (AC (10mg kg^{-1}) N=12) post-injection and the % sucrose intake and total volume drunk calculated.

Forced swim test (FST): The FST was performed using group 4 (Fig. 1) based on previously described methods [26]. Following handling and habituation on day 1, mice (N=8 per treatment) were placed in a transparent glass vessel (25 cm in height, 14 cm in diameter) filled with 10 cm of water at 24 ± 2 °C on day 2 and activity recorded using Ethovision software. Activity was manually scored 0.5h post-injection during the last 5 min of a single 6 min test session for escape behaviours (active swimming) and non-escape behaviours (small movements only to keep the head above water and immobility).

Novel object recognition (NOR): The NOR test was performed using group 5 (Fig. 1) using the same apparatus and conditions as used for the OFT and performed over two consecutive days as previously described. On day 1, mice (vehicle N=9; AC N=10) were handled and habituated to the testing environment for 15 min. For the sample phase on day 2, mice placed in the centre of the OF box and initially allowed to explore two identical objects for 10 min, with the time spent exploring the two identical objects recorded using Ethovision software (Noldus Information Technology, Netherlands) and scored by hand. The mouse was then returned to its home cage and injected with the relevant treatment. The choice phase was performed 0.5h later, with the mouse returned to the centre of the OF box and allowed to explore the two objects for 10 min, with one of the initial objects being replaced by a novel object which the mouse had not seen previously. The time spent exploring the novel object compared to the familiar object was scored by hand and the discrimination ratio (time exploring novel object/ total object exploration time x 100) calculated.

Electrophysiology

Male and female C57BL/6J mice (N=8) were euthanized by cervical dislocation and immediately decapitated. Brains were rapidly removed and transferred to ice-cold oxygenated (95% O2; 5% CO2) sucrose-based artificial cerebro-spinal fluid (ACSF) solution containing (in mM): sucrose 50, NaCl 87, $NaHCO_3$ 25, KCl 3, NaH_2PO_4 1.25, $CaCl_2$ 0.5, $MgCl_2$ 3, sodium pyruvate 3 and glucose 10. Coronal brain sections (300 µm) containing the lateral habenula (LHb) were then prepared using a Leica VT1200S vibratome (Leica Biosystems, UK). Following sectioning, slices were incubated in oxygenated sucrose ACSF at 35 °C for 30 min, and then incubated for a further 30 min at room temperature in ACSF containing (in mM) NaCl 115, $NaHCO_3$ 25, KCl 3, NaH_2PO_4 1.25, $CaCl_2$ 2, $MgCl_2$ 1, sodium pyruvate 3 and glucose 10. Following the incubation period, slices were stored at room temperature in oxygenated ACSF for ≥1 h before recording. Individual slices were transferred to a submerged recording chamber and continually perfused with oxygenated ACSF at a flow rate of 2–3 ml / min and visualized with a Luigs and Neumann LN-Scope System (Luigs and Neumann, Germany). Glass micropipettes (5-8 MΩ) were filled with an intracellular solution containing (in mM:) potassium gluconate 125, HEPES 10, KCl 6, EGTA 0.2, $MgCl_2$ 2, Na-ATP 2, Na-GTP 0.5, sodium phosphocreatine 5, with the pH adjusted to 7.2 with KOH. Visually guided whole-cell patch clamp recordings were obtained from LHb neurons that were acquired between 10-25 kHz and low pass-filtered between 2-10 kHz using an ITC-18 digitizer interface (HEKA, Germany), with data acquired using Axograph X software (www.axograph.com). Once in whole-cell configuration, spontaneous action potential (AP) firing was recorded in current-clamp at the resting membrane potential with neurons with AP firing >0.5 Hz considered to be spontaneously active, whereas neurons with AP firing <0.5 Hz were deemed silent (Han et al., 2020). Spontaneous excitatory postsynaptic currents (sEPSCs) were recorded in voltage clamp at −70 mV following characterization of neuron type in current clamp. All data was analysed offline using Axograph X.

Microglial isolation and culture

Microglia were isolated and cultured as described previously [27]. Briefly, brains from male C57/Bl6J mice (12 weeks old, N=4) were isolated by terminally anaesthetizing with 3% isoflurane (33.3% O_2 and 66.6% N_2O) and transcardial perfusion with ice-cold 0.9% NaCl. Brains were immediately placed into ice-cold HBSS (ThermoFisher, UK) and minced using a 22A scalpel before centrifugation (300 x g, 2

min) and digestion using the MACS Neural Dissociation Kit (Miltenyi Biotec, UK) according to manufacturer's instructions. Briefly, brain tissue was incubated in enzyme P (50 μL/brain) diluted in buffer X (1900 μL/brain) for 15 min at 37 °C under gentle rotation before addition of enzyme A (10 μL/brain) in buffer Y (20 μL/brain) and further incubation for 20 min at 37 °C under gentle rotation. Following digest tissue was dissociated mechanically using a Dounce homogenizer (loose Pestle, 20 passes) on ice and centrifuged (400 x g, 5 min at 4 °C). To remove myelin, tissue was resuspended in 35% isotonic Percoll (GE Healthcare, UK) overlaid with HBSS and centrifuged (800 x g, 40 min, 4 °C). Following centrifugation, the supernatant and myelin layers were discarded and the pellet resuspended in MACS buffer (PBS, 0.5% low endotoxin BSA (Sigma-Aldrich, UK), 2 mM EDTA, 90 μL/brain). Anti-CD11b microbeads (Miltneyi Biotec, UK) were added (10 μL/brain) and the suspension incubated for 15 min at 4 °C before running through pre-rinsed (MACS buffer) LS columns attached to a magnet (Miltneyi Biotec, UK). After washing with 12 mL MACS buffer columns were removed from the magnet and cells retained (microglia) were flushed in 5 mL MACS buffer. Microglia were resuspended in DMEM/F-12 (Thermo Fisher Scientific, UK) supplemented with 1% PenStrep, 10% FBS, 500 pg/mL rhTGFβ-1 (Miltneyi Biotec, UK), 10 pg/μL mCSF1 (R&D Systems, UK). Microglia were counted using a haemocytometer and plated out at 40,000 cells/well onto a flat-bottom TC-treated 96-well plate (Corning, UK) coated with poly-L-lysine. Cells were cultured for 7 days at 37°C in a humidified incubator under 5% CO_2 with a half media change on day 3.

Quantitative real-time PCR

C57BL/6J male mice (10-12 weeks old) were given a single injection of either AC (100 mg kg^{-1}) or vehicle and the cerebellum, hippocampus and hypothalamus collected 2 hours (N=5 per group) or 24 hours (N=5 per group) post injection and stored in RNAlater tissue storage solution (Life Technologies, UK) at <4 ^{o}C until processing. Total RNA was isolated from the brain regions and reverse transcribed, with cycle threshold (C_t) values generated as described previously (Abulkassim et al, 2016). qRT-PCR results were analysed using the relative quantification method of comparative Ct (ΔΔCt), with β-actin acting as the calibrator or 'housekeeping' gene. Expression of the calibrator genes was stable and did not differ significantly between control and treatment groups (data not shown). All primer sequences and RT-PCR methods were as previously described (Abulkassim et al, 2016) with reactions run in triplicate and the mean Ct value from the three reactions was used for subsequent data analysis.

ELISA

Serum: C57BL/6J male mice were euthanised by cervical dislocation at 2 and 24 hours post vehicle (N=13) and AC (100 mg kg^{-1}, 2h N=10, 24h N=10) injection and blood harvested immediately. Serum was isolated from whole blood samples and cytokine levels determined by ELISA, with IL-1β (#88-7013-88), IL-6 (#88-7064-88), TNF-α (#88-7324-88) and IFN-γ (#88-7314-88) levels determined according to manufacturer's instructions (ThermoFisher, UK).

Spleen and lymph nodes: C57BL/6J male mice (N=5) were euthanised as described above and spleens and lymph nodes harvested. Single cell suspensions were cultured in 24-well plates at 2 X 10^6 cells mL^{-1} and exposed to AC (50 µM) or vehicle controls. Supernatants were collected after 2 and 24 h, with IL-1β, IL-6, TNF-α and IFN-γ levels determined using ELISA kits as described above.

Microglia: All cell stimulations were performed after 7 days in culture, with microglia exposed to either AC (50 µM), lipopolysaccharide (LPS; 100 ng mL^{-1}) or vehicle control for 2 or 24h by direct addition into culture medium. Following cell stimulation, supernatant was removed and analysed for IL-1β (#DY401-05), IL-6 (#DY406-05) and TNF-α (#DY410-05; all R&D Systems, UK).

Data analysis and statistics

All data are expressed as mean ± S.E.M. and N = the number of animals used and n= number of LHb neuronal recordings with normality of all data sets determined with Shapiro–Wilk tests. For the electrophysiological experiments, no difference in AC effect was found between sexes, so data for both sexes was combined. Data were then compared, with tests used highlighted in the relevant figure legend, by unpaired or paired two-tailed Student's t tests, one-way analysis of variance with either a Dunnett's or Bonferroni post hoc test or two-way repeated-measures ANOVA with a Bonferroni post hoc test as appropriate using GraphPad Prism (v5.0 or v9.0) or Minitab (v19.0). Differences were considered significant when $P < 0.05$.
