# Supplementary material for: Protease-activated receptor 2 activation induces behavioural changes associated with depression-like behaviour through microglial-independent modulation of inflammatory cytokines
Source: Psychopharmacology (Berl). 2021 Dec 9;239(1):229–42. doi: 10.1007/s00213-021-06040-1 (PMC8770450; doi:10.1007/s00213-021-06040-1)
Supplement: Supplementary file 2 — Supplementary file2 (PDF 48 KB) [file 213_2021_6040_MOESM2_ESM.pdf]

Protease-activated receptor 2 activation induces depression-like behaviour through microglial-independent modulation of inflammatory cytokines.

Serge Moudio, Ashleigh Willis, Karolina Pytka, Roua Abulkassim, Ros R. Brett, Jack F. Webster, Christian Wozny, Mark Barbour, Hui-Rong Jiang, David G Watson, Josie C. van Kralingen, Scott M. MacKenzie, Michael Daniels, Barry W McColl, Sandra Sossick, Hugh N Nuthall & Trevor J. Bushell.

ESM2 AC selectivity

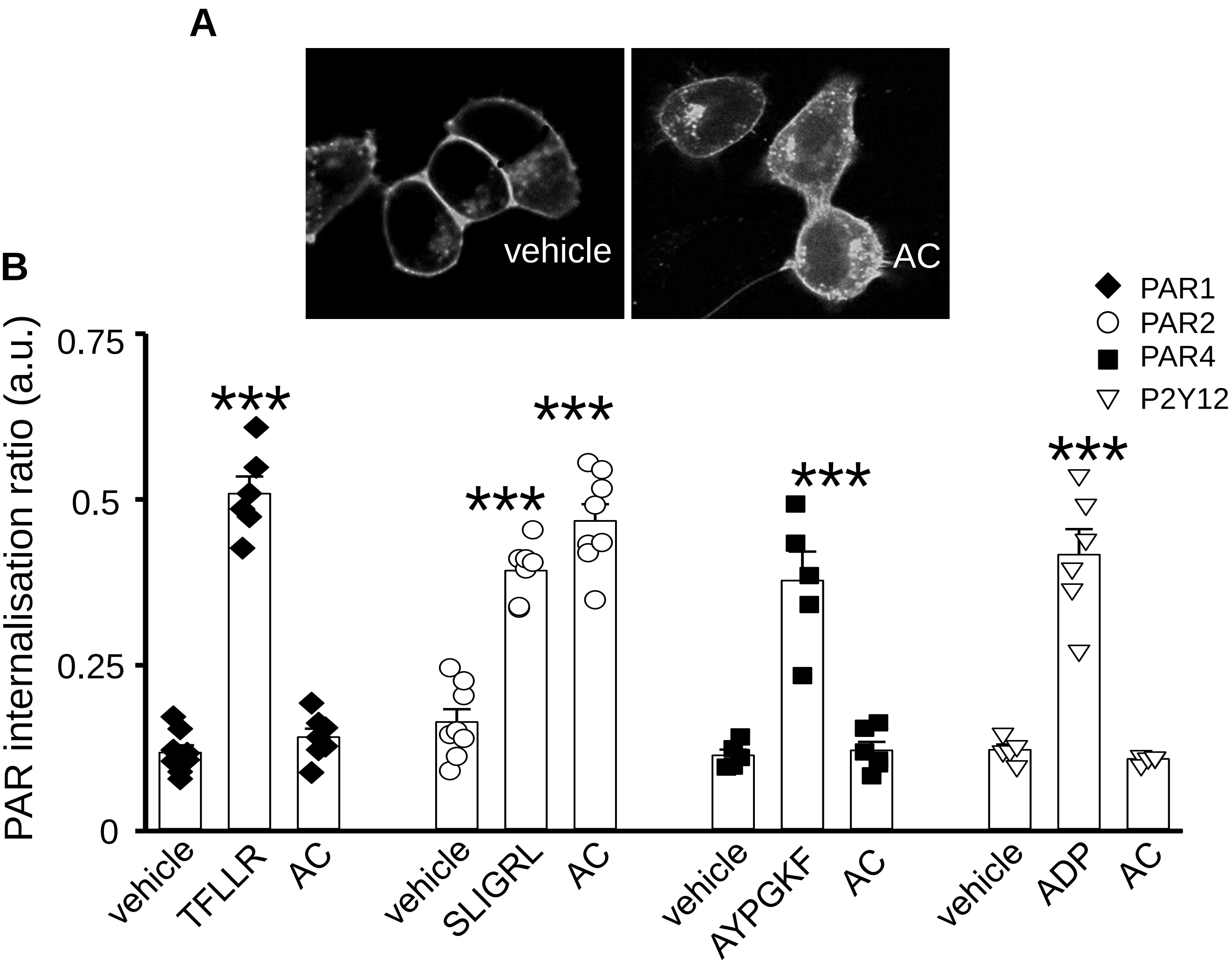

ESM2: AC selectively activates PAR2. **a** Representative images revealing that AC (50 µM) internalises PAR2-GFP when compared to vehicle control. **b** Summary bar chart confirming that AC selectively induces PAR2 internalisation over other PARs and P2Y12, with the function of all GPCRs confirmed using selective activators.
